# Factors Predicting Effectiveness of Neoadjuvant Therapy for Esophageal Squamous Cell Carcinoma

**DOI:** 10.1097/MD.0000000000003365

**Published:** 2016-04-18

**Authors:** Yu Ohkura, Masaki Ueno, Toshiro Iizuka, Shusuke Haruta, Tsuyoshi Tanaka, Harushi Udagawa

**Affiliations:** From the Department of Gastroenterological Surgery (YO, MU, SH, TT, HU); and Gastroenterology (TI), Toranomon Hospital, Tokyo, Japan.

## Abstract

The aim of the study was to elucidate pretreatment factors that can predict the outcome of neoadjuvant chemoradiotherapy or chemotherapy (NAC(R)T) and help us choose treatment strategies appropriate for individual patients.

Few studies have investigated whether clinical data obtainable before the treatment can predict the efficacy of NAC(R)T.

Of 1540 patients treated for esophageal squamous cell carcinoma (ESCC) at our department between January 2000 and June 2014, those who underwent surgical resection of cStage II or more advanced ESCC after NAC(R)T (113 NACRT and 146 NACT patients) were enrolled in this study. Information all available before the treatment was analyzed to extract factors that can predict the effectiveness of NAC(R)T. NAC(R)T was considered effective when Grade 2 or greater treatment efficacy was achieved based on the histological grading system.

NACRT was effective in 51 (45%) of 113 patients. The analysis of 35 pretreatment factors showed that female sex (hazard ratio [HR] = 3.650; 1.181–11.236), absence of dyslipidemia (HR = 3.284; 1.341–8.041), and histologically poorly differentiated tumor (HR = 2.431; 1.052–5.619) were factors predicting NACRT effectiveness. On the other hand, NACT was effective in 21 (14%) of 146 patients. The analysis of pretreatment factors showed that absence of dyslipidemia (HR = 10.204; 1.302–83.33) and therapy with docetaxel, cisplatin, and 5-fluorouracil (HR = 2.097; 1.027–4.280) were factors predicting NACT effectiveness.

The findings of this study investigating factors that could predict the outcome of NAC(R)T suggest that the prevalence of dyslipidemia influences the outcome of NAC(R)T for ESCC.

## INTRODUCTION

Esophageal squamous cell carcinoma (ESCC) is the most dangerous form of gastrointestinal cancer.^1,2^ As the first report of its efficacy in the 1980s,^3–6^ chemoradiotherapy (CRT) has been used widely to treat ESCC. Findings from the histopathological examination of esophageal cancer resected after CRT are extremely useful in judging CRT efficacy and selecting additional therapies and consequently in predicting the prognosis.^7,8^ Several studies investigated the predictors of post-CRT prognosis,^9–11^ whereas others analyzed pretreatment imaging findings and histopathological findings to reveal the predictors of CRT efficacy.^12–16^ As in CRT, several studies reported the efficacy of neoadjuvant chemotherapy (CT), making it standard treatment for esophageal cancer.^17–21^ However, except the studies using pretreatment imaging findings, no studies used pretreatment factors such as patient background and hematological findings to predict the outcomes of NAC(R)T. The discovery of pretreatment clinical or biopsy findings that can predict or enhance the efficacy of CRT or CT would enable us to establish more effective and individualized treatment strategies. In this study, we assessed the independent factors that can predict the outcome of NAC(R)T (hereinafter, CRT/CT-effectiveness) applied to the patients with ESCC.

## MATERIAL AND METHODS

### Patients

Of 1540 patients treated for esophageal cancer at our hospital between January 2000 and June 2014, 113 patients with cStage II or above ESCC who had undergone CRT as the first therapy and then esophageal resection were enrolled in this study. Neoadjuvant CT regimens in the CRT protocol were high-dose FP (800 mg/m^2^ 5-fluorouracil [FU], 80 mg/m^2^ cisplatin) in 28 patients; low-dose FP (200 mg/m^2^ 5FU, 4 mg/m^2^ cisplatin) in 66; and docetaxel, cisplatin, and 5-fluorouracil (DCF) (60 mg/m^2^ docetaxel, 50 mg/m^2^ cisplatin, 500 mg/m^2^ 5FU) in 19. The dose of radiation was >50 Gy and ≤50 Gy in 11 and 102 patients, respectively. Data obtainable before therapy were analyzed to extract factors predicting CRT effectiveness.

Similarly, 146 patients who had undergone esophageal resection after NACT were analyzed to reveal factors that can predict efficacy specific to CT. Neoadjuvant CT regimens in this group were high dose FP (800 mg/m^2^ 5FU, 80 mg/m^2^ cisplatin) in 95 patients; DCF (60 mg/m^2^ docetaxel, 50 mg/m^2^ cisplatin, 500 mg/m^2^ 5FU) in 38; and FAP (600 mg/m^2^ 5FU, 30 mg/m^2^ doxorubicin, 60 mg/m^2^ cisplatin) in 13.

### Methods

The analysis items were 35 factors (10 pre-treatment patient background factors, 18 pre-CRT biochemical factors, 5 tumor factors, and 2 treatment factors) and 33 factors in total (10 pre-treatment patient background factors, 18 pre-CT biochemical factors, 4 tumor factors, and 1 treatment factor) were analyzed to extract factors predicting NACRT and NACT effectiveness, respectively. In our hospital, the tumor grading was performed in accordance with American Joint Committee on Cancer (AJCC) guidelines.^22^ C(R)T was considered to be effective when the resected specimen was diagnosed to show Grade 2 or greater treatment effect by histopathological assessment. The histopathological effects of NAC(R)T was defined according to the Japanese Classification of Esophageal Cancer, 10th edition.^23^ The grading systems—Grade 0: ineffective, no recognizable cytological or histological therapeutic effect. Grade 1: slightly effective, apparently viable cancer cells account for 1/3 or more of the tumor tissue, but there is some evidence of degeneration of the cancer tissue or cells. Grade 1a: viable cancer cells accounting for 2/3 or more tumor tissue. Grade 1b: viable cancer cells accounting for 1/3 or more, but <2/3, of tumor tissue. Grade 2: moderately effective, viable cancer cells account for <1/3 of the tumor tissue, whereas the other cancer cells are severely degenerated or necrotic. Grade 3: markedly effective, no viable cancer cells are evident.^23^ In this study, the routine pathology was performed by multiple pathologist. However, in our hospital, a single chief pathologist reviewed all pathological materials and routinely made a definitive diagnosis of the histopathological response grade 0 to 3 of NACRT or NACT. This study was approved by the Institutional Review Board of our hospital.

### Statistics

Cumulative overall survival (OS) was analyzed by the Kaplan–Meier method. A difference between 2 groups was analyzed using the Chi-square and Mann–Whitney U tests, and multiple regression analysis was used to reveal factors predicting C(R)T effectiveness. All variables with significance of *P* < 0.10 in the simple Cox proportional hazards models were entered into multiple Cox proportional hazards models. In multiple Cox proportional hazards models, *P* < 0.05 was considered significant. Statistical analysis was performed using the SPSS ver.19 (SPSS Inc., Chicago, IL), with significance set at *P* < 0.05. Staging was performed in accordance with the Union for International Cancer Control TNM Classification of Malignant Tumors (version 7).^24^

## RESULTS

### Overall Survival of Patients who Underwent NAC(R)T

**FIGURE 1:**
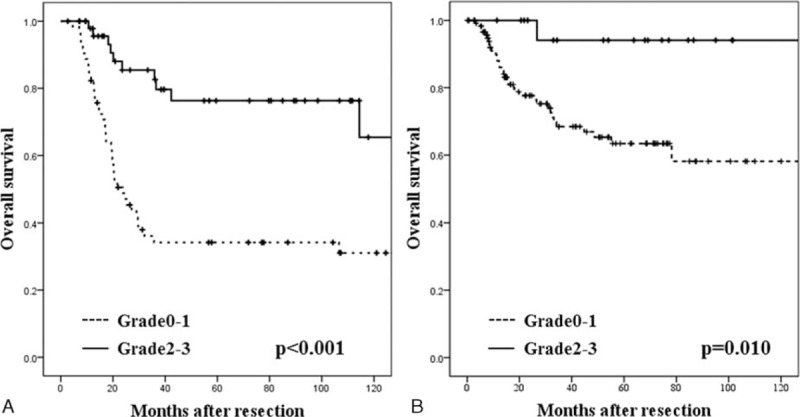
(A) Survival curves of patients who underwent NACRT. Actuarial survival rates of patients with a histological grade 2 or greater were significantly (*P* *<* 0.001) higher than those of patients with a histological grade of 0–1. (B) Survival curves of patients who underwent NACT. Actuarial survival rates of patients with a histological grade 2 or greater were significantly (*P* *=* 0.010) higher than those of patients with a histological grade of 0–1. NACRT *=* neoadjuvant chemoradiotherapy, NACT *=* neoadjuvant chemotherapy.

The overall survival curves of patients who underwent NACRT or NACT are shown in Figure 1. NAC(R)T, when resulted in downstaging due to tumor shrinkage and achieved Grade 2 or greater treatment efficacy based on the histological grading system, increasing survival rates were revealed. The actuarial survival rates of patients who underwent NACRT with a histological grade 2 or greater (5-year survival, 76 %) were significantly (*P* *<* 0.001) higher than those of patients with a histological grade of 0–1 (5-year survival, 34 %). On the other hand, the actuarial survival rates of patients who underwent NACRT with a histological grade 2 or greater (5-year survival, 94 %) were significantly (*P* *=* 0.010) higher than those of patients with a histological grade of 0 to 1 (5-year survival, 63 %).

### Factors Predicting NACRT Effectiveness

We first examined 113 patients who had undergone NACRT at our hospital and revealed that 51 (45 %) patients had achieved Grade 2 or greater treatment efficacy using the histological grading system for post-treatment evaluation. The univariate analysis between 62 patients with a histological grade of 0 to 1 and 51 patients with a grade of 2 or greater showed a significant difference in 3 factors: female sex, absence of dyslipidemia, and poorly differentiated tumor (Table 1). Patients had been defined as having dyslipidemia when hematological findings showed ≥140 mg/dL of LDL-cholesterol, <40 mg/dL of HDL-cholesterol, or ≥150 mg/dL of triglycerides.^25,26^ This study included patients who had been undergoing drug therapy for dyslipidemia.

**TABLE 1:**
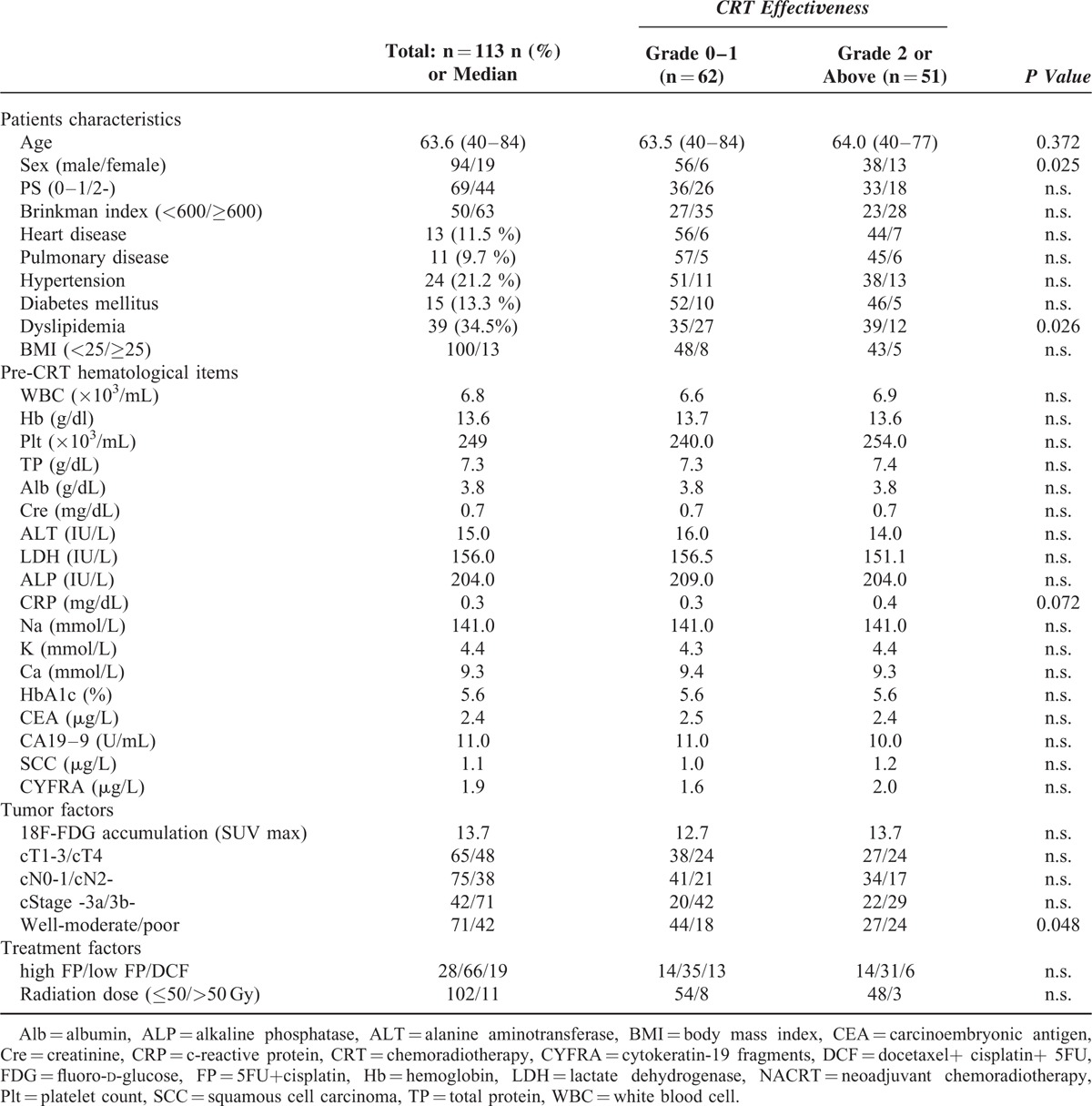
Patient Characteristics and the Results of Univariate Analysis of the Factors Predicting NACRT Effectiveness

Multivariate analysis was performed using the results of the univariate analysis (Table 2). The selected variables were age, female sex, CRP value, absence of dyslipidemia, and poorly differentiated tumor. It revealed that female sex (male/female = 94/19, HR = 3.650), absence of dyslipidemia (positive/negative = 39/74, HR = 3.284), and histologically poorly differentiated tumor (well to moderately/poorly differentiated = 71/42, OR = 2.431) were all independent factors predicting NACRT effectiveness.

**TABLE 2:**
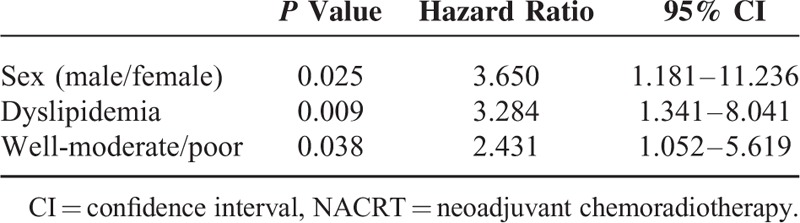
Results of Multivariate Analysis of the Factors Predicting NACRT Effectiveness

### Factors Predicting NACT Effectiveness

We then examined 146 patients who had undergone NACT at our hospital and revealed that 21 (14 %) patients had postoperatively achieved Grade 2 or above treatment efficacy. The univariate analysis between 125 patients with a histological grade of 0–1 and 21 patients with a grade of 2 or above showed a significant difference in 2 factors: absence of dyslipidemia and DCF therapy (Table 3).

**TABLE 3:**
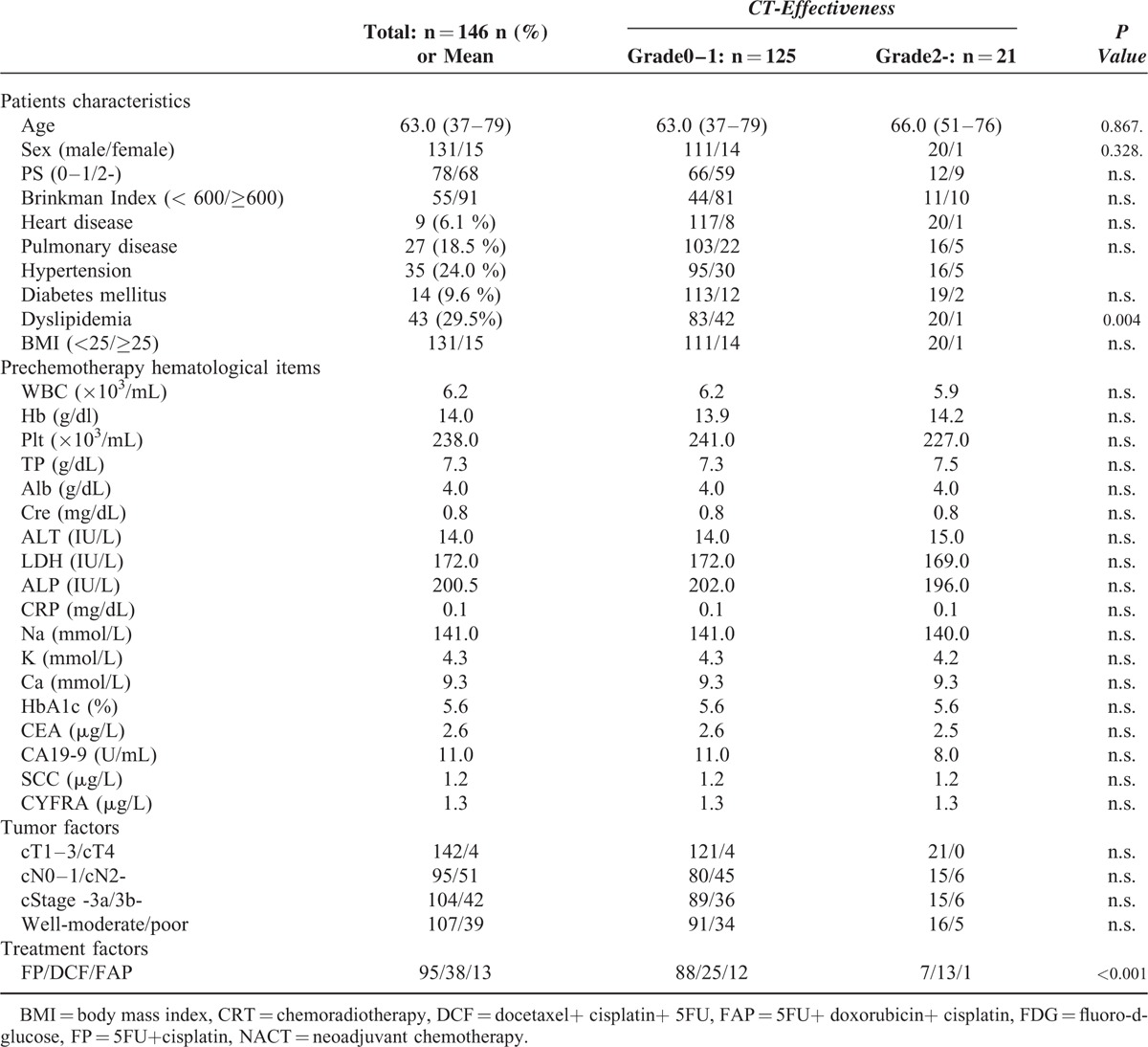
Patient Characteristics and the Results of Univariate Analysis of the Factors Predicting NACT Effectiveness

Multivariate analysis was performed using the results of the univariate analysis (Table 4). The selected variables were age, sex, absence of dyslipidemia, and DCF. It showed that absence of dyslipidemia (positive/negative = 43/103, HR = 10.204) and DCF (HR = 2.097) were both independent factors predicting NACT effectiveness.

**TABLE 4:**
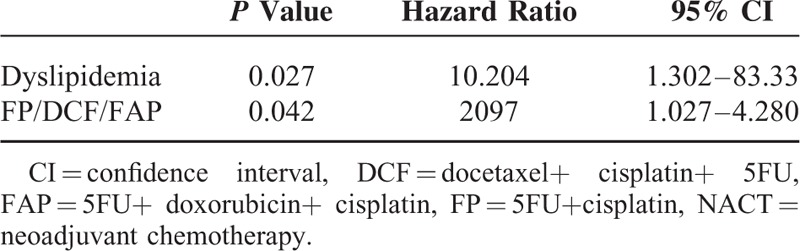
Results of Multivariate Analysis of the Factors Predicting NACT-Effectiveness

## DISCUSSION

In general, a postoperative increase in survival rates among patients treated with NACRT or NACT is attributed to the tumor-shrinking effect of NAC(R)T.^4–11^ In a study of esophageal resection after CRT, Swisher et al reported that 3-year survival rates were significantly higher among patients who had smaller remnant tumors in postoperative histopathological examination.^8^ In addition, Law et al showed that pN0, female sex, and R0 were statistically significant good prognostic factors.^9^ According to Schneider et al^10^ and Okumura et al,^11^ after R0 resection surgery, prognosis was significantly better among patients with shrunken tumors or with N0 classification. In the present study also, NAC(R)T, when resulted in downstaging due to tumor shrinkage and achieved Grade 2 or greater treatment efficacy based on the histological grading system, increasing survival rates were revealed. These findings therefore showed that it is highly feasible to predict the outcome of NAC(R)T preoperatively. If such prediction is possible, we can select therapy that is more suitable to each patient. In this study, we therefore investigated pretreatment factors associated with the tumor-shrinking effects of NAC(R)T and revealed that factors predicting NACRT effectiveness were female sex, absence of dyslipidemia, and histologically poorly differentiated tumor, whereas factors predicting NACT effectiveness were the absence of dyslipidemia and DCF therapy.

To date, several studies have reported imaging findings as the predictors of the effectiveness of CRT.^11–16^ According to Owaki et al, endoscopic ultrasound performed before and early after CRT predicted treatment outcome accurately with sensitivity of 85 % and specificity of 95 %, which was comparable with histopathological evaluations.^12^ In addition, Okumura et al performed preoperative combination examination of esophagography and endoscopy and showed that combination examination predicted histopathological outcomes with ≥80% accuracy.^13^ However, these studies predicted histopathological treatment outcome using imaging data obtained before and after CRT, but real prediction based solely on patient background factors, biochemical data, or preoperative evaluation items which are available before the treatment is started, has not been performed. Other studies performed positron emission tomography-computed tomography before treatment to show the correlation between the accumulation of fluoro-D-glucose (FDG) and the histological grading of treatment outcome.^13–16^ However, no correlation between the preoperative accumulation of FDG and histopathological findings was observed in the database at our hospital. In this study, we therefore used only pretreatment factors such as patient background, pre-CRT biochemical data, and tumor and treatment factors to reveal factors associated with Grade 2 or above treatment outcome. As shown in Tables 1 and 2, female sex, poorly differentiated tumor, and absence of dyslipidemia were the factors predicting the efficacy of CRT.

However, no previous study has shown that CRT is more effective in female than in men. In this study, the rate of smoking was significantly lower in female patients than in male patients (*P* < 0.001), and first of all, this might have been a reason for female sex being a significant factor even though the Brinckman index which was included in the initial patient factors was not the factor predicting the CRT effectiveness. The Brinckman Index was obtained using the following equation: number of cigarettes smoked per day × number of years smoked.^27^ Smoking is known to induce systemic hypoxia which decreases the radiosensitivity of cells as well as tumors.^28–34^ In addition, nicotine was reported to induce radioresistance.^35^ Therefore, a higher smoking rate in our male patients than in female patients may have contributed to the suppression of CRT efficacy because of a reduction in radiosensitivity due to hypoxia and an increasing radioresistance due to nicotine. Second, female hormone estrogens have beneficial effects on plasma lipid and lipoprotein concentrations and reduce arteriosclerosis extent on a number of animal models.^36–38^ Although the arteriosclerotic disease generally increase significantly after menopause, the incidence of arteriosclerotic disease among female was much lower until at least age 75.^39,40^ In this study, the age of the patients were around 60; therefore, the risk of arteriosclerosis of the female patients is lower than male patients. A relation between arteriosclerosis and treatment resistance is indicated on the following paragraph. In this regard, however these 2 reasons are only a hypothesis thoroughly. If we can accumulate more cases, a more precise analysis might show strong similarities.

The Bergonie–Tribondeau's law, which was proposed by Bergonie and Tribondeau in 1906, states that the sensitivity toward radiation is high among tissues with a large proportion of morphologically and functionally undifferentiated cells, high mitotic activity, and a long and active developmental stage.^41^ Since then, many studies have investigated radiosensitivity in humans.^42–44^ In the present study, we revealed poorly differentiated tumor as a factor significantly influencing the efficacy of CRT. Poorly differentiated cells have high mitotic activities compared with well-differentiated ones, and tumors containing a large proportion of poorly differentiated cells have a high degree of malignancy but are more susceptible to radiation.

A novel finding of this study is that the prevalence of dyslipidemia affects the efficacy of NAC(R)T. We can guess arteriosclerosis are strongly related between the efficacy of NAC(R)T and dyslipidemia. In the past reports, it is well known that the arteriosclerosis is strongly associated with dyslipidemia.^45–47^ As arteriosclerosis progresses, tumor tissue becomes more hypoxic and phenotypically more malignant, enhancing its resistance toward CT and CRT.^29–34^ Basic research showed that the adaptor protein p66Shc, which is normally located adjacent to the insulin receptor, plays a significant role in dyslipidemia and arteriosclerosis and, through its expression, increases cellular resistance toward radiotherapy and chemotherapy.^48,49^ This suggests that patients with dyslipidemia develop resistance toward radiotherapy and chemotherapy, thereby compromising the tumor-shrinking effect of C(R)T, because of tumor hypoxia enhanced by arteriosclerosis and the expression of p66Shc. Our study is the first clinical, but not basic, study to report similar findings.

The limitation of this study was possible bias in the selection of surgery and neoadjuvant therapy because this was a retrospective study of NAC(R)T. The treatment applied is rather heterogeneous. NACT is the standard treatment for advanced esophageal carcinoma (e.g., cStage II or above) in our hospital. On the other hand, there is a tendency to indicate NACRT in patients with relatively advanced cancer (particularly, in T factors). In fact, NACRT is an accepted standard of treatment with locally advanced esophageal cancer. In addition, this study enrolled patients with esophageal cancer who had successfully undergone surgery after NACRT, indicating that these patients had relatively good general conditions and cancer had progressed only up to a certain point. Furthermore, at our hospital, we tend to select DCF therapy for patients with highly advanced cancer, indicating that clinical stage is less severe in patients undergoing NACT with FP than those undergoing NACT with DCF. Finally, no review pathology was performed, but routine pathology assessed in this study.

## CONCLUSIONS

The findings of this study revealed that female sex, absence of dyslipidemia, and poorly differentiated tumor are the predictors of NACRT efficacy, with the absence of dyslipidemia also being a significant factor for NACT. The elucidation of clinicopathological factors that can predict the outcome of neoadjuvant therapy will help us establish a more effective treatment plan for each patient.
